# Prenatal diagnosis using genetic sequencing and identification of a novel mutation in MMACHC

**DOI:** 10.1186/s12881-015-0196-8

**Published:** 2015-07-07

**Authors:** Yanan Zong, Ning Liu, Zhenhua Zhao, Xiangdong Kong

**Affiliations:** Center of Prenatal Diagnosis, the First Affiliated Hospital of Zhengzhou University, Zhengzhou, 450052 People’s Republic of China

**Keywords:** Combined methylmalonic aciduria and homocystinuria, *MMACHC* gene, Gene mutation, Prenatal diagnosis

## Abstract

**Background:**

Combined methylmalonic aciduria and homocystinuria, cobalamin(cbl)C deficiency, is a rare disorder of intracellular vitamin B_12_(cbl) metabolism caused by mutations in the *MMACHC* gene. Both genetic and biochemical approach have been established to diagnose children and fetuses with *cblC* deficiency, while in China there is no report of prenatal genetic diagnosis of *cblC* deficiency. The aim of the present study was to characterize the mutational spectrum of *cblC* deficiency and investigate the feasibility of genetic-sequencing-based prenatal diagnosis for *cblC* deficiency.

**Methods:**

10 pedigrees were recruited in this study with the probands clinically and biochemically confirmed combined methymalonic aciduria and homocystinuria. Peripheral blood samples were collected for *MMACHC* genetic test from the probands and their parents (4 probands had already dead) and 50 control subjects. The entire coding region and adjacent splice sites of *MMACHC* were sequenced. After the genotypes of the pedigrees were identified, chorionic villi sampling were performed for 3 high-risk pregnant women for prenatal genetic diagnosis.

**Results:**

A total of 7 mutations were identified: c.217C > T (R73X), c.394C > T (R132X), c.463G > C (G155R), c.609G > A (W203X), c.616C > T (R206W), c.658-660delAAG (220delK), and c.567dupT (I190YfsX13), as well as 2 polymophsims: c.321G > A(V107V), c.-302G > T. And G155R is a novel mutation that haven’t been reported in the literatures. All the 6 probands identified with compound heterozygous mutations or homozygous mutations of *MMACHC* gene, and all the parents of the probands were found to have one *MMACHC* mutation at a heterozygous level. Prenatal diagnosis of fetuses from 3 families with a child affected *cblC* deficiency showed that one fetus had the same compound heterozygous mutations as the proband, one did not have *MMACHC* mutation, and the third fetus had a mutation at a heterozygous level of *MMACHC* gene. Results from the follow-ups were consistent with the prenatal diagnosis.

**Conclusion:**

A novel mutation p.G155R of the *MMACHC* gene is identified. Genetic diagonsis is an accurate and convenient method for prenatal diagnosis and early intervention of combined methylmalonic aciduria and homocystinuria.

## Background

Methylmalonic acidemia (MMA), the most common inborn disorder of organic acid metabolism, is inherited as an autosomal recessive disease caused by defects of methylmalonyl CoA mutase (MCM) or disorders of intracellular cobalamin metabolism [1]. MMA can be classified as isolated methymalonic acidemia and combined methylmalonic acidemia and hyperhomocysteinemia based on biochemical features. Combined methymalonic aciduria and homocystinuria consists of four subtypes, *MMACHC(cblC), MMADHC* (*cblD combined*, *cblD-MMA* and *cblD-HCY*)*, LMBRD1(cblF)* and *ABCD4(cblJ)* [2]. All of them may cause the deficiency of methylcobalamin (MeCbl) and adenosylcobalamin (AdoCbl) and then reduce the activity of methionine synthase and methylmalonyl CoA mutase (MCM). Thus both methylmalonic acid and homocysteine will be accumulated in the blood and urine [3, 4]. In China, 80 % of patients with MMA are combined methylmalonic acidemia and hyperhomocysteinemia [5], among which the *cblC* type is the most common. The gene associated with the *cblC* defect is *MMACHC* (OMIM 277400), which maps to chromosome region 1p34.1 and consists of 5 exons and expressing a 5.2 kb mRNA encoding a 282 amino acid protein [6]. To date, 77 mutations have been identified in this gene, and genotype-phenotype correlations have been studied [7–10]. Based on the age at onset, two forms of *cblC* deficiency have been described [3, 4]. Early-onset patients present in the first year of life with feeding difficulties, hypotonia, developmental delay, seizures, pigmentary retinopathy and anemia. Late-onset patients exhibit ataxia, dementia, psychosis and other neurologic symptoms after 4 years old [1, 3].

With the application of tandem mass spectrometry and gas chromatography–mass spectrometry (GC-MS), more and more affected children have benefitted from early diagnosis and treatment, greatly reducing mortality and morbidity in children with MMA. Although MMA is a partially treatable disorder, both the high mortality during the acute phase and the chronic damage to the nervous system, will lower the quality of patient’s life and increase the family economic burden. The most effective intervention is to avoid the birth of children with MMA through prenatal diagnosis. Many successful techniques for prenatal diagnosis of combined methymalonic aciduria and homocystinuria have been reported in China and other countries, including measuring the activity of methylmalonyl-CoA mutase in the amniotic fluid, amniotic fluid cells and chorionic cells and quantification of cbl metabolites in amniotic fluid cells [11, 12]. In recent years, analysis of acylcarnitine analysis by electrospray tandem mass spectrometry(ESI/MS/MS) has been used in prenatal diagnosis. A combination of genetic and biochemical analyses have been reported, while in China there is no report of prenatal diagnosis through simple genetic diagnosis. In the present study, *MMACHC* mutations were analyzed in 10 pedigrees of combined methymalonic aciduria and homocystinuria (*cblC* type) and a novel mutation was identified. Prenatal genetic diagnosis was performed for 3 families.

## Methods

### Patients

Ten pedigrees were recruited for the study which came to our hospital, the Third Affiliated Hospital of Zhengzhou University, and the Children's Hospital of Zhengzhou from March 2011 to April 2013. There was no consanguinity among the children, and none of their parents was consanguineous marriage. Children from 6 families were directly diagnosed with combined methymalonic aciduria and homocystinuria based on clinical features. For the other four families, the probands were diagnosed postmortem (with complete clinical information), because the four probands presented severe symptoms within 3 months and died within 1 years old.

All patients showed different levels of respiratory infections, feeding difficulties, malnutrition, seizures, drowsiness, seizures, hypotonia, and psychomotor retardation. Urinary organic acid analysed by gas chromatography/mass spectrometry (GC-MS,QP2010, Schimadzu, Japan) showed high levels of methylmalonic acid. Serum total homocysteine was elevated determined by fluorescence polarization immunoassay (IMX automatic analyzer,Abbott, the USA) [13].

This study was approved by the Medical Ethics Committee in the First Affiliated Hospital of Zhengzhou University. All of the analyzed samples were obtained with the parents’ informed consent.

### DNA extraction

2 ml peripheral venous blood was taken from the 6 probands and 10 couples of parents, and then anticoagulated by ethylenediaminetetraacetic acid disodium salt (EDTAK_2_). The transabdominal chorionic villi sampling were performed with ultrasonic guidance for the high-risk women at 10 to 13 ^+ 6^ weeks of gestation. Genomic DNA was extracted from peripheral blood samples and chorionic villi samples using the DNA extraction kit (TIANGEN Biotech, Beijing, China) according to the manufacturer’s instructions.

### PCR reactions and DNA sequencing

The coding exons and the flanking introns of the *MMACHC* gene were amplified by polymerase chain reaction (PCR). The PCR primers were described by Lerner-Ellis *et al.* [6] (Table 1). The reaction mixture of a final volumn of 25 μL contained 10 × PCR buffer,2.5 mM dNTP, 0.2 μM of each primer, 1U Taq enzyme, and 50 ng DNA template. The amplification program consisted of an initial denaturation at 94 °C for 3 min, followed by 30 cycles at 94 °C for 30s, 57- 62 °C for 40s, and 72 °C for 50s, with a final extension at 72 °C for 8 min. PCR products were checked on a 2 % agarose gel and then purified. The direct sequencing was performed with an ABI3130xl gene analyzer (Life Technologies, USA) using the ABIBigDye3.1 sequencing kit (Life Technologies, USA) according to manufacturer’s instructions. Both strands of the shifted exons were sequenced. Sequences were aligned and inspected to identify nucleotide variations using a reference sequence from Ensemble (NM_015506).

**Table 1:** PCR primers for mutation analysis of *MMACHC*

Primers	Gene fragments	Sequence	Length
MMACHC-1 F:	Exon 1	GGGATACCGTGATGATACGC	680 bp
MMACHC-1R:		GAACCCAGGAGGATCAGAGG	
MMACHC-2 F:	Exon 2	TGCATCACATAGCGTCAGTG	467 bp
MMACHC-2R:		AGCCTGGCTTTAGGGTATCA	
MMACHC-3 F:	Exon 3	TCATGTTTTCCCTTCTGAGGA	395 bp
MMACHC-3R:		CAAAGCTAATTTGTTCTGGGTTG	
MMACHC-4 F:	Exon 4	AGGCCTAGCTTGCAATGATG	694 bp
MMACHC-4R:		GAAGGCAGATGGGAATTCTG	

### Bioinformatics analysis of sequence variations

By searching the HGMD database, Exome Variant Server and ExAC browser, the new mutations were identified and the frequency of each known variantion is available. The analysis software ClustalW was used to compare the homologous sequences of human *MMACHC* with other species. PROVEAN prediction and Polymorphism Phenotyping (PolyPhen) were performed to analyze the impact of new mutations. The corresponding fragments of *MMACHC* from 50 unrelated healthy individuals were amplified and sequenced.

### Prenatal diagnosis

After the genotype of the probands and their parents were identified, prenatal genetic diagnosis was performed for the 3 fetuses at 10 to 13 ^+ 6^ weeks’ gestation. DNA was extracted from chorionic villi samples and mutations of the *MMACHC* gene were detected. To exclude contamination from the mother, the PowerPlex 16 HS System kit (Promega, Madison, WI, USA) were used and the results were analyzed using ABI3130xl (Life Technologies, USA) and the software GeneMapper v3.2. The evaluation criteria for absence of contamination were defined as follows: the fluorescence peaks of the gene-sites of the fetus were also appeared in its parents, and there is only one fluorescence peak acquired from its mother. The prenatal genetic diagnosis will be carried out within 1 week.

### Follow-up

Specimens of umbilical cord blood or fibroblast cells from the three fetuses were collected for genetic diagnosis. Organic acid levels of urine and serum total homocysteine were detected for two newborns at 1 month old.

## Results

### Analysis of MMACHC mutations

**Fig. 1:**
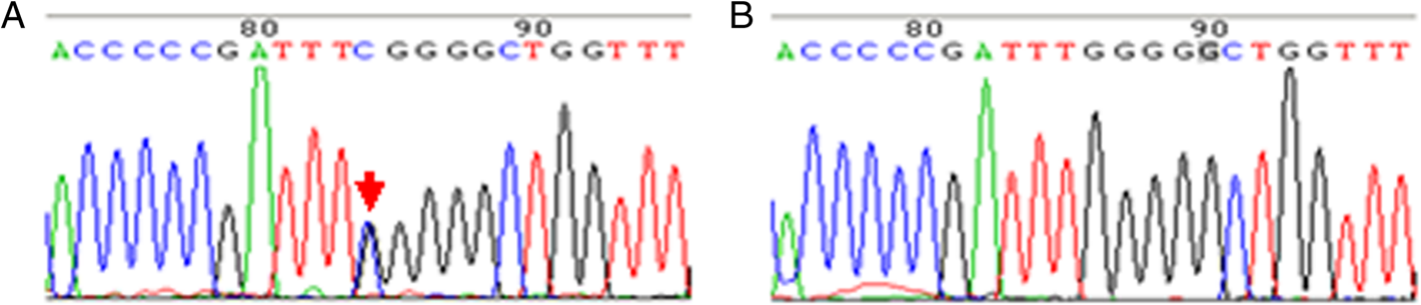
DNA sequencing maps. **a** a positive sequencing of DNA of the probands which shows G / C heterozygous peaks indicating c.463G > C (G155R) heterozygous mutation; **b** DNA sequencing of the normal families which shows homozygous peaks. The position with detected mutation is indicated with arrow

**Fig. 2:**
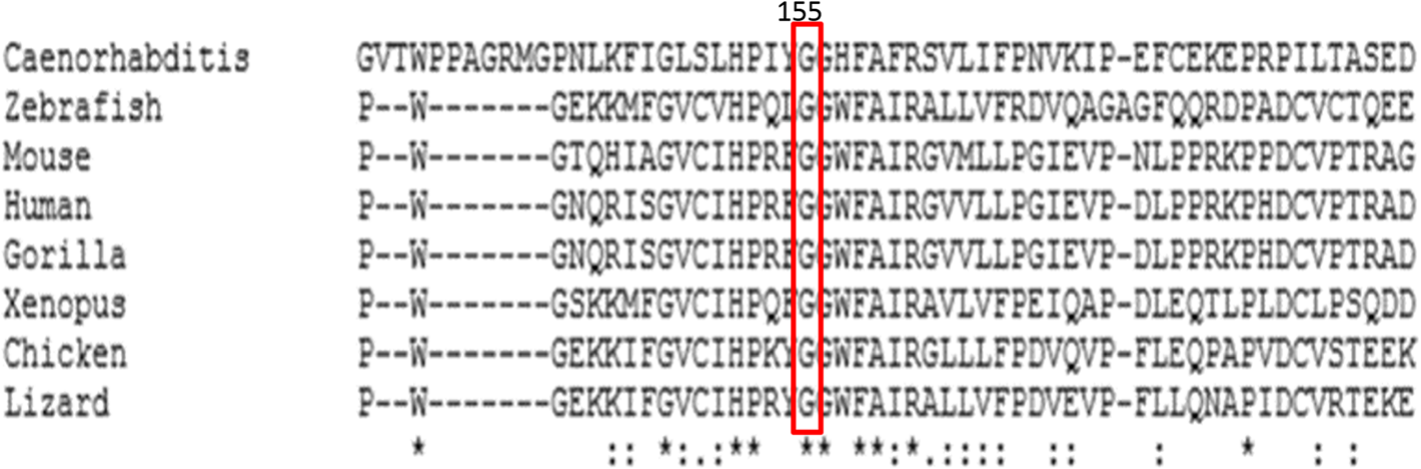
MMACHC protein sequences from different organisms. The G155 in MMACHC is highly conserved between humans, gorillas, zebrafish, rodents, and lizards. Amino acid region 139-188 of human protein sequences are shown

Nine vatiations in *MMACHC* were found in the 10 pedigrees (Table 2), two of them were silent gene polymorphisms, c.321G > A(V107V), c.-302G > T. Mutations of c.217C > T, c.394C > T and c.609G > A were nonsense which resulted in premature termination of translation at amino acid residue arginine (73), cysteine (132) and tryptophan (203), respectively. The missense mutation c.463G > C led to the replacement of glycine (155) with arginine (Fig. 1). The insertion in c.567dupT resulted in a frameshift mutation starting from isoleucine (190). The deletion mutation of c.658 ~ 660delAAG led to the deletion of lysine during protein translation. The missense mutation c.616C > T may cause the replacement of arginine (206) with tryptophan. None of the mutations listed here were found in exon sequencing of the *MMACHC* gene from the 50 control subjects. By searching the ExAC browser and other databases, the p.G155R mutation have not been reported before, thus it is considered to be a novel mutation. Comparative analysis of the amino acid sequence of MMACHC from human, chimpanzee, zebrafish, rodents, and lizards was performed, and we found that the residue G155 is highly conserved between various species (Fig. 2) indicating it might be essential for the normal function of *MMACHC*. Another type of mutation has been previously reported as pathogenic at the same codon, c.464G > A, p.G155E [14]. Besides the p.G155R mutation was considered pathogenic as PolyPhen2 software predicted that the functional consequences was probably damaging (score of 1.000). In addition, the PROVEAN score of the p.G155R mutation was –7.167 (cutoff:–2.5).

**Table 2:** Mutational analysis, clinical data and prenatal diagnosis of combined MMA and HC families

No.	Proband	Mutation Maternal	Mutation Paternal	Onset age	Clinical data			Follow-up	Fetus Genotype
					HC (μM)	MMA (μM)	Ocular abnormalit-ies		
								(age and clinical details)	
1*#	/	220delK	W203X	1 months	43	20.8	NA.	Dead of severe malnutrition at 6 months old	W203X, 220delK
2	R132X	W203X	R132X	6 months	99	187.3	Normal^	1.5y,developmental delay HC decreased to 60 μM	
	W203X								
3	G155R	G155R	c.567dupT	1 month	NA	75.1	nystagmus	MMA decreased to 24.6 μM	
	c.567dupT								
4#	/	W203X	220delK	3 weeks	NA.			Dead at 1 month old	
5#	/	R73X	220delK	2 months	NA.			Dead at 8 month old	
6*	R206W	R206W	W203X	40 days	158	45.1	nystagmus	3y,developmental delay,	R206W,
	W203X							HC, MMAare normal	——
7	W203X	W203X	220delK	10 days	131	958.9	nystagmus	developmental delay, malnutrition	
	220delK								
8*	W203X	W203X	W203X	2 weeks	174	215.2	Normal^	15 months, taking medicine promptly and properly for a period of time, the patient has being in good recovery	——,
	W203X								——
9#	/	220delK	W203X	NA.	NA.			Dead at 4 months old	
10	W203X	W203X	W203X	3 months	108	29.4	Visual inattention nystagmus	6 months, Can’t raise his head, developmental delay	
	W203X								

Note: *indicates prenatal diagnosis families; # indicates the proband in the family was dead; / indicates that the genotype of the dead proband was not available; ——indicates no mutation was detected; ^ indicates the proband’s parents said it’s normal without test

HC:value of serum homocysteine (reference value:5-15 μM)

MMA:value of urine methylmalonic acid (reference value:0.2-3.6 μM)

NA.:not avaliable,bacause the patient is from other hospital or can’t get through telephone

The variation c.-302 T > G located in the 5’ untranslated region, and c.321G > A (V107V) was a synonymous mutation, both of them are known polymorphisms.

### Prenatal diagnosis of MMACHC gene and follow-up

In family 1, the mother of one deceased proband was found to have 220delK heterozygous mutation and the father had W203 heterozygous mutation. The fetus was identified with compound heterozygous mutation of 220delK/W203X, and was affected *cblC* deficiency. In family 6, the proband had compound heterozygous mutations of R206W/W203X,while the mother had R206W mutation and the father had W203X mutation respectively. The fetus was identified as a heterozygous carrier of R206W mutation. In family 8, the proband had W203X mutations at a homozygous level, and both the parents had one W203X allele, while the fetus did not have any *MMACHC* mutations (Table 2). Chorionic villi tissues collected from the fetuses of were analyzed to exclude maternal contamination using the PowerPlex 16 HS System kit, thus the results of gene detection in this study was reliable.

After genetic counseling, family 1 chose to terminate the pregnancy. The fetal fibroblast cells were collected for genetic test, and the result were consistent with that of the prenatal diagnosis. Family 6 and 8 chose to continue the pregnancies, and the umbilical cord blood was taken for gene diagnosis after the childbirth. The results were consistent with that of prenatal diagnosis. Organic acid levels of urine and serum total homocysteine of the two newborns were normal at 1 month old.

## Discussion

In this study, 9 variations in *MMACHC* were identified in 10 pedigrees with *cblC* deficiency including a novel mutation, G155R. By transabdominal chorionic villi sampling and DNA sequencing, genetic prenatal diagnose is performed and proved to be accurate and convenient. Among 3 cases of prenatal diagnosis, one fetus was found to be affected by *cblC* deficiency with compound heterozygous mutations of *MMACHC*, one fetus was determined to be a mutation carrier, while the third fetus had a normal genotype. Genetic sequencing after birth for the second and the third babies confirmed the results of prenatal diagnosis.

To date, 77 diferent mutations in the *MMACHC* gene have been reported. Among these mutations, several mutations are common but ethnic-specific. The c.271dupA mutation is the most common allele in Europeans, with a mutation frequency of 40 %, and ususlly occurred in early-onset cases. The c.394C > T mutation at a homozygous level occurred mostly in Native American and Middle Eastern patients, and is associated mostly with a late-onset disease [8, 9]. The mutation c.609G > A (W203X) occurred most frequently in patients in East Asia [15–17].

*Han* [16] reported that 156 mutations were found in 158 disease alleles in 79 Chinese patients with combined methymalonic aciduria and homocystinuria. The mutations c.609G > A (W203X), c.658_660delAAG (K220del), c.482G > A (R161Q), c.394C > T (R132X), and c.80A > G (Q27A) were the most frequent and accounted for 80 % of all mutations. Among these 5 mutations, the c.609G > A mutation was the most common, accounting for 50 %, followed by the c.658_660delAAG mutation, which accounted for 9 %. In this study, 10 c.609G > A mutations were found in 20 alleles accounting for 50 % of the total mutations, which further confirmed that the c.609G > A mutation in *MMACHC* was the hot spot in Chinese patients. Another recent study proposed that the c.609G > A mutation may be specific to East Asian populations [10].

A total of seven mutations and two polymorphisms were detected in this study. The mutations c.217C > T, c.394C > T and c.609G > A lead to the encoding of a stop codon at amino acid residue 73,132 and 203 resulting in premature termination of translation. The c.658_660delAAG mutation, located in the C-terminal region of *MMACHC,* causes deletion of lysine at residue 220. The c.616C > T (R206W) mutation is first reported in China, and its function have not been reported so far. The c.567dupT mutation causes a frameshift at codon 189 and a premature termination at codon 202. The c.463G > C (G155R) mutation is reported for the first time although c.464G > A (p.G155E) mutation at the same codon has been reported previously [14]. The *MMACHC* glycine 155 is highly conserved among many species and PROVEAN and PolyPhen analysis for p.G155E suggested pathogenic. Among seven mutations found in this study, six were located in exon 4. Liu *et al.* [15] showed that exon 3 and exon 4 are mutation hot spots. Therefore genetic screening of *cbl* deficiency may be performed preferentially in exon 3 and exon 4 of *MMACHC*.

In general, late-onset patients have better survival and response to treatment compared with early-onset patients. As an early-onset patient, patient 6 in this study had been given treatment with hydroxycobalamin, carnitine, betanine and folinic acid, as well as rehabilitation programs promptly after diagnosis. During follow-up at 3 years old, we found that his urine levels of MMA decreased to normal and plasma levels of homocystenuria decreased to 10.6 μmol/l. The result confirmed the importance of early intervention of the disease.

In this study, 3 families with probands affected the early-onset *cbl* deficiency underwent prenatal genetic diagnosis. W203X mutation was found at a homozygous level in one family, and at a heterozygous level for the other two families. *Wang* reported that in 31 cases of early-onset cbl C deficiency, 25 (80.6 %) had the W203X mutation, indicating that the W203X mutation may be associated with early-onset phenotype [16]. However, more data are needed to confirm this correlation.

The early-onset *cbl* deficiency is a severe neonatal genetic disease. Although the outcome will be better through early detection and treatment, most children die before one year old. Prenatal diagnosis is helpful and necessary to parents who want a second child. In families with deceased probands, genetic diagnosis can be made by testing the parental *MMACHC* genes. If both of the parents have a *MMACHC* mutation at a heterozygous level, the proband’s genotype can be deduced. Prenatal diagnosis for *cbl* deficiency is sought for terminating an affect pregnancy, instituting treatment during pregnancy or alleviating mental pressure of the couples and so on. Prenatal OHCbl administration may reduce the maternal metabolites, however the complications of *cbl* deficiency such as retinopathy could not be ameliorated [3]. In this study, chorionic villi samples were collected in the first-trimester pregnancy for prenatal genetic diagnosis for 3 families. The fetus of the first family was identified with 220delK/W203X compound heterozygous mutation and the parents chosed terminate the pregnancy. After abortion the fibroblast cells of that fetus were tested for *MMACHC* mutations, and the results were consistent with the prenatal diagnosis. The fetus of the second family had R206W mutation at a heterozygous level, while the third fetus was normal. After genetic counseling, the two families decided to continue their pregnancies. After the babies were born, they were determined to be healthy by physical examination and laboratory tests. There are several approaches to make biochemical prenatal diagnosis of MMA [17]. Reaserchers could determine the methylmalonate in amniotic fluid(AF) and maternal urine, measure the mutase reaction and cobalamin metabolism in cultured amniocytes, study the [^14^C]-propionate incorpation, analysis acylcarnitine, and assay the MCM activity in chorionic villi(CV) [18]. There seems to be a lack of consensus on the most reliable method, and preferred sample on which to conduct these investigations. And it is reported that the results could be false nagtive/positive [2, 17]. Although the metabolic analysis of amniotic fluid will be carried out simply, the molecular genetic testing is the best method to make prenatal diagnose in the first trimister,since amniocentesis shouldn’t be performed before the 16^th^ gestational week to avoid complications. Our success with prenatal genetic diagnosis of *cblC* deficiency shows that it is acurate and economical. It could also be an effective method of genetic screening for affected families. But the information of the proband or its parents must be avaliable, and maternal contamination of the DNA sample should be excluded by marker analysis. Preimplantation genetic diagnosis could also be considered for the families whose pathogenic mutation have been identified.

## Conclusions

We present the molecular confirmation of infantile onset cobalamin C type-combined methylmalonic acidemia and homosystinuria in 6 probands and their parents, as well as 4 additional couples with diseased children affected *cblC* deficiency, and a novel mutation of the *MMACHC* gene, p.G155R is identified. We also performed prenatal diagnosis using CVS(chorionic villi sampling) and DNA sequencing for three high-risk pregnant women successfully.
